# Factors for Assessing the Effectiveness of Early Rehabilitation after Minimally Invasive Total Knee Arthroplasty: A Prospective Cohort Study

**DOI:** 10.1371/journal.pone.0159172

**Published:** 2016-07-13

**Authors:** Tetsuya Amano, Kotaro Tamari, Shigeharu Tanaka, Shigehiro Uchida, Hideyuki Ito, Shinya Morikawa, Kenji Kawamura

**Affiliations:** 1 Department of Physical Therapy, Faculty of Health and Medical Sciences, Tokoha University, Hamamatsu, Shizuoka, Japan; 2 Graduate School of Health Science, KIBI International University, Takahashi, Okayama, Japan; 3 JICA Guatemala Office, Zona, Calle, Guatemala; 4 Department of Physical Therapy, Kawasaki Junior College of Rehabilitation, Kurashiki, Okayama, Japan; 5 Department of Rehabilitation, Faculty of Rehabilitation, Hiroshima International University, Higashihiroshima, Hiroshima, Japan; 6 Department of Physical Therapy, Yamaguchi Allied Health College, Yamaguchi, Yamaguchi, Japan; 7 Department of Rehabilitation, Hohsyasen Daiichi Hospital, Imabari, Ehime, Japan; Harvard Medical School/BIDMC, UNITED STATES

## Abstract

The effectiveness of current rehabilitation programs is supported by high-level evidence from the results of randomized controlled trials, but an increasing number of patients are not discharged from the hospital because of the schedule of the critical path (CP). The present study aimed to determine which factors can be used to assess the effectiveness of early rehabilitation. We enrolled 123 patients with medial knee osteoarthritis (OA) who had undergone unilateral minimally invasive total knee arthroplasty for the first time. The following factors were assessed preoperatively: the maximum isometric muscle strength of the knee extensors and flexors, maximum knee and hip joint angle, pain, 5-m maximum walking speed, sex, age, body mass index, exercise habits, Kellgren-Lawrence grade, femorotibial angle, failure side (bilateral or unilateral knee OA), and functional independence measure. We re-evaluated physical function (i.e., muscle strength, joint angle, and pain) and motor function (5-m maximum walking speed) 14 days postoperatively. Changes in physical function, motor function (5-m maximum walking speed), and number of days to independent walking were used as explanatory variables. The postoperative duration of hospitalization (in days) was used as the dependent variable in multivariate analyses. These analyses were adjusted for sex, age, body mass index, exercise habits, Kellgren-Lawrence grade, femorotibial angle, failure side, and functional independence measure. The duration of hospitalization was significantly affected by the number of days to independent walking (p < 0.001, β = 0.507) and a change in the 5-m maximum walking speed (p = 0.016, β = -0.262). Multiple regression analysis showed that the radiographic knee grade (p = 0.029, β = 0.239) was a significant confounding factor. Independent walking and walking speed recovery were considered to reduce the duration of hospitalization. Therefore, these indices can be used to assess the effectiveness of early rehabilitation.

## Introduction

Evidence is objective information used to make a clinical decision, and evidence-based medicine is recommended in the clinical setting. In Japan, a critical path (CP) that incorporates evidence-based treatment was introduced in the late 1990s, and it has been effective for promoting a team approach to medical care and enhancing patient satisfaction [1]. Total knee arthroplasty (TKA) is recommended as an effective and cost-efficient treatment for patients with knee osteoarthritis (OA) who have significant pain, limited range of motion, and decreased muscle strength [2]. Additionally, preliminary research regarding the implementation of the CP after TKA has shown that it reduces the duration of hospitalization and associated medical costs [3, 4]. Furthermore, muscle strength reinforcement and range of motion exercises (primarily for the repaired knee) are effective for improving function after TKA [5–8], and are commonly performed during postoperative rehabilitation.

Although the effectiveness of current rehabilitation programs is supported by high-level evidence from randomized controlled trials (RCTs), an increased number of patients are not discharged from the hospital because of the schedule of the CP. RCTs are an excellent method for obtaining a high level of clinical evidence. However, because RCTs use strict selection criteria to enhance quality, some patients deviate from these criteria, which may be a limitation of this study design. Recently, minimally invasive total knee arthroplasty (MI-TKA) has been associated with a decrease in the duration of hospitalization. Compared to conventional TKA, MI-TKA has been reported to reduce postoperative pain and to significantly improve the recovery of joint function [9–11]. Moreover, a study of patients who underwent MI-TKA reported that the patients’ condition was satisfactory at 5 years postoperatively [12]. Although MI-TKA is effective for reducing the duration of hospitalization, the efficacy of early postoperative rehabilitation interventions has not been studied. The present study aimed to determine which factors can be used to assess the effectiveness of early rehabilitation.

## Materials and Methods

### Patients

**Fig 1:**
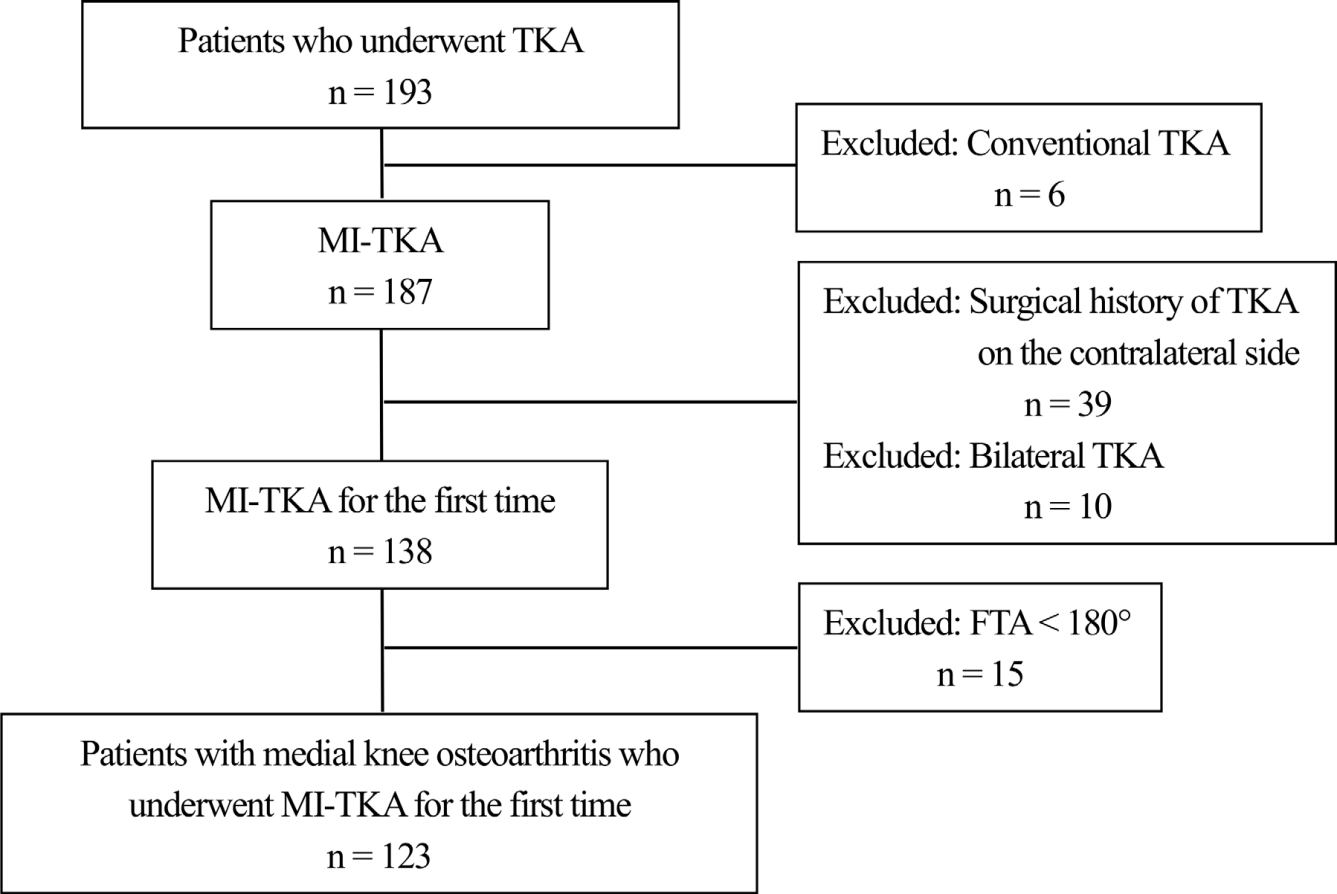
Flowchart of patients. MI-TKA, minimally invasive total knee arthroplasty; TKA, total knee arthroplasty; FTA, femorotibial angle.

This study included data from 4 hospitals that had introduced the CP. We evaluated 193 patients with knee OA who had undergone TKA between July 2013 and December 2015 at the Kurashiki Central Hospital, Yamaguchi Grand Medical Center, Kawasaki Medical School Hospital, and Kurashiki Medical Center. The inclusion criteria were patients between the ages of 50 and 90 years who had been diagnosed with Kellgren-Lawrence (KL) grade 3 or 4 knee OA. Exclusion criteria were patients who underwent conventional TKA, those who had a surgical history of TKA on the contralateral side, patients who underwent TKA bilaterally, and those who had a femorotibial angle (FTA) <180° (Fig 1). Therefore, 123 patients (22 men, 101 women) with a mean age of 75.5 years (range, 53–86 years) were evaluated. Patients’ demographic and medical characteristics are presented in Table 1. We evaluated their demographic characteristics (sex, age, body mass index [BMI], and exercise habits) and medical characteristics (KL grade, FTA, failure side [bilateral or unilateral knee OA], and functional independence measure [FIM]). Patients with exercise habits were defined as those who exercised for ≥ 30 minutes twice per week (walking, strength training, and/or stretching) for ≥ 1 year. The failure side was defined as bilateral or unilateral knee OA.

**Table 1:** Patients’ baseline demographic and medical characteristics.

Characteristic		
Sex, n (%)	Men: 22 (18)	Women: 101 (82)
Age (years)	75.5 ± 6.6	
Body mass index (kg/m^2^)	25.4 ± 3.7	
Exercise habits, n (%)	Yes: 39 (32)	No: 84 (68)
Kellgren-Lawrence grade, n (%)	Grade 4: 72 (59)	Grade 3: 51 (41)
Femorotibial angle (°)	186.8 ± 5.1	
Failure side, n (%)	Bilateral knee OA: 80 (65)	Unilateral knee OA: 43 (35)
Functional independence measure (points)	120.8 ± 4.4	

Data are presented as mean ± standard deviation or n (%).

The study design was approved by the Institutional Review Board of KIBI International University (no. 14–31). Participants gave written informed consent before data collection began.

### Design

In this observational study, TKA involved replacing the entire knee with a prosthesis. The mini-midvastus, mini-subvastus, and mini-medial parapatellar approaches for TKA were used. Seventeen surgeons performed the surgeries in the current study. All patients were treated as per the CP shown in Table 2. The 4 study hospitals began rehabilitation on the first postoperative day, and it was performed at bedside (depending on the patient’s condition) for 20–40 minutes/day, and in the rehabilitation room for 40 minutes/day. These interventions were similar to previously reported interventions; no special procedures were performed. Physical therapy and assessments were implemented by 25 physical therapists at the study hospitals. Power analyses were performed using G*Power 3 (Heinrich-Heine University, Düsseldorf, Germany) [α error, 0.05; statistical power, 0.80; effect size, f^2^ = 0.15; number of predictors, 10]. The required sample size in this study was calculated as more than 118 participants. We confirmed that the number of patients enrolled in this study was sufficient.

**Table 2:** Rehabilitation schedule for the critical path.

Rehabilitation schedule	
Postoperative day 1	Rehabilitation starts at bedside using standing and transfer exercises, range of motion exercises, and muscle strengthening exercises
Postoperative day ≥2	Rehabilitation starts in the rehabilitation room using walking exercises with parallel bars and a walker
Postoperative day ≥3	T-cane walking exercises start
Postoperative day ≥7	Patient begins walking up and down stairs
Postoperative day ≥14	Patient is discharged with rehabilitation instructions

The research variables were measured prior to surgery and on postoperative day 14. The following factors were assessed preoperatively: physical function (maximum isometric muscle strength of the knee extensors and flexors, maximum knee and hip joint angles, and pain) and the 5-m maximum walking speed (5mMWS) as an indicator of motor function. Patients’ physical function (i.e., muscle strength, joint angle, and pain) and motor function (5mMWS) were re-assessed on postoperative day 14. In addition, the number of days required for independent walking was assessed for each patient.

Maximum isometric muscle strength was measured using a hand-held dynamometer (HHD) [μTas F-1; Anima Corp., Tokyo, Japan] while the patients were seated with their knees flexed at 90° relative to the thigh. The HHD sensor was positioned on the distal front of the lower leg during knee extension and on the distal rear of the lower leg during knee flexion. A belt was used to secure the sensor to the part of the body that was being evaluated, and it was fixed to a table leg during the knee extension measurement as well as around the tester’s lower leg during the knee flexion measurement [13]. The measurements were repeated twice with a break between each measurement, and the mean value was used to calculate the torque: body weight ratio (Nm/kg). Pain of the knee joint on the operated side was evaluated during bed rest by using a numerical rating scale (NRS) that ranged from 0 (no pain) to 10 (worst pain imaginable). The 5mMWS assessment was an in-room test that consisted of an 11-m linear walking path with 3-m preliminary paths on both ends. The 5mMWS assessment started when the patient’s lower limb crossed the start line (i.e., the end of the first 3-m preliminary path), and it ended when the patient crossed the end line (i.e., the line separating the end of the 5-m path from the second 3-m preliminary path). The number of days to independent walking was defined as the period the patient required to achieve independent walking using a T-cane. The following conditions had to be met: 1) patients achieved independent walking (2 physical therapists observed the walking, and if patients were able to walk for >50 m with a T-cane, independent walking was achieved), 2) patients expressed confidence in their ability to walk with the T-cane, and 3) patients achieved a timed up and go test (TUG) result of <13.5 seconds [14]. The duration of hospitalization was recorded as the number of postoperative hospitalization days.

### Data analysis

Multiple regression analysis was used to determine which factors can be used to assess the effectiveness of early rehabilitation. The explanatory variables were defined as changes in the physical function, motor function, and days to independent walking, whereas the dependent variable was defined as postoperative hospitalization days. The magnitude of change in the explanatory variables was calculated as positive if they improved from baseline to postoperative day 14, and it was defined as negative if the 14-day postoperative functions were inferior to the preoperative functions. Explanatory variables were entered into the multiple regression model using the stepwise method. Data were analyzed using multivariate analysis, which was adjusted for sex, age, BMI, exercise habits, KL grade, FTA, failure side, and FIM. KL grade 4 was assigned a score of 1, and grade 3 was assigned a score of 0. In accordance with previous research [15, 16], each explanatory variable was subjected to an initial screening of its relationship with the dependent variable by using univariate analysis. The level of significance was set at p < 0.20 to ensure that the explanatory variables were included at this stage. Then, significant explanatory variables were entered into the regression analysis model. SPSS software version 22.0 (IBM, Tokyo, Japan) was used to analyze the collected data, and differences were considered statistically significant at a p-value <0.05.

## Results

Preoperative function, function at postoperative day 14, and days to independent walking are presented in Table 3.

**Table 3:** Preoperative and postoperative function results.

Assessment	Before surgery	Postoperative day 14
Knee extensor strength on the operated side (Nm/kg)	0.81 ± 0.34	0.49 ± 0.23
Knee extensor strength on the non-operated side (Nm/kg)	0.92 ± 0.36	0.83 ± 0.31
Knee flexion strength on the operated side (Nm/kg)	0.43 ± 0.20	0.30 ± 0.14
Knee flexion strength on the non-operated side (Nm/kg)	0.49 ± 0.22	0.46 ± 0.20
Maximum hip extension angle on the operated side (°)	11.7 ± 8.6	11.8 ± 8.2
Maximum hip extension angle on the non-operated side (°)	12.3 ± 8.1	12.9 ± 7.9
Maximum knee extension angle on the operated side (°)	-9.2 ± 8.2	-6.7 ± 6.4
Maximum knee extension angle on the non-operated side (°)	-6.3 ± 7.3	-5.0 ± 6.2
Maximum knee flexion angle on the operated side (°)	121.9 ± 17.2	112.2 ± 16.2
Maximum knee flexion angle on the non-operated side (°)	129.9 ± 14.2	129.7 ± 13.4
NRS (points)	2.2 ± 2.7	1.7 ± 1.8
5mMWS (m/s)	1.00 ± 0.33	0.89 ± 0.29
Days to independent walking	13.8 ± 4.3	

NRS, numeric rating scale; 5mMWS, 5-m maximum walking speed.

Data are presented as mean ± standard deviation.

**Fig 2:**
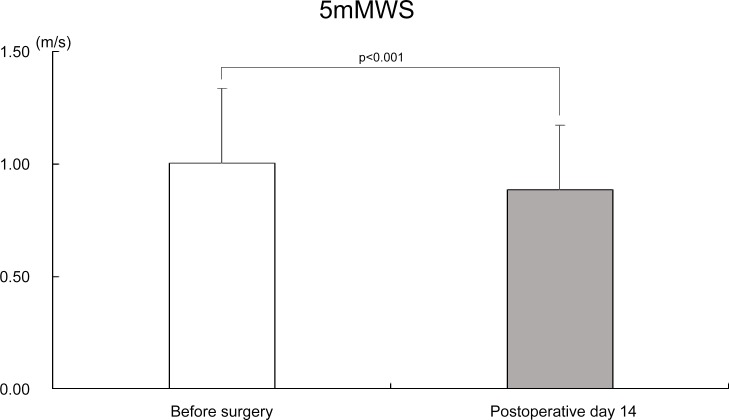
Bar chart showing five-meter maximum walking speed before surgery and at postoperative day 14. 5mMWS, 5-m maximum walking speed. The error bars show the standard deviation.

Results from the univariate analysis showed that change in the knee extensor strength on the operated side, change in the knee flexion strength on the operated side, change in the maximum hip extension angle on the non-operated side, change in the maximum knee extension angle on the operated side, change in the maximum knee extension angle on the non-operated side, change in the maximum knee flexion angle on the operated side, change in the NRS, change in the 5mMWS, and days to independent walking were significantly associated with postoperative hospitalization days (p < 0.20). In multivariate analysis (p < 0.001, R = 0. 568, R^2^ = 0.323), the factors affecting the number of postoperative hospitalization days were the number of days to independent walking (p < 0.001, β = 0.528) and change in the maximum hip extension angle on the non-operated side (p = 0.024, β = -0.204) (Table 4). Results that included the confounding factors (p < 0.001, R = 0.711, R^2^ = 0.505) show that the number of days to independent walking (p < 0.001, β = 0.507) and the magnitude of change in the 5mMWS (p = 0.016, β = -0.262) significantly affected the number of postoperative hospitalization days (Table 4). These results indicate that patients who exhibited minimal recovery in terms of their 5mMWS at postoperative day 14 and those who experienced a delay in achieving independent walking would be more likely to experience prolonged hospitalization. The average time to independent walking was 13.8 ± 4.3 days. The mean change in the 5mMWS was -0.15 ± 0.30 m/s, and the changes at postoperative day 14 did not exceed the preoperative results (Fig 2). In addition, the radiographic knee grade (p = 0.029, β = 0.239) was a significant confounding factor (Table 4); 72 patients had a KL grade of 4, and 51 had a KL grade of 3. Thus, the number of days to independent walking and walking speed are factors that can be used to assess the effectiveness of early rehabilitation, and they were independent of the effect of confounding factors.

**Table 4:** Multiple regression analysis of factors that can be used to assess the effectiveness of early rehabilitation.

Independent variable	β	p-value	95% confidence interval
Without confounding factors			
Intercept	-	<0.001	11.694–16.845
Days to independent walking	0.528	<0.001	0.362–0.725
Change in the maximum hip extension angle on the non-operated side	-0.204	0.024	-0.404 to -0.030
With confounding factors			
Intercept	-	0.200	-84.051–18.008
Days to independent walking	0.507	<0.001	0.308–0.747
Change in the 5mMWS	-0.262	0.016	-7.222 to -0.769
Sex	-0.098	0.356	-3.510–1.283
Age	0.186	0.080	-0.015–0.250
BMI	0.087	0.491	-0.185–0.380
Exercise habits	-0.087	0.412	-2.879–1.196
KL grade	0.239	0.029	0.221–3.963
FTA	0.068	0.536	-0.128–0.244
Failure side	0.040	0.721	-1.751–2.517
FIM	0.168	0.130	-0.058–0.444

BMI, body mass index; KL grade, KL, Kellgren-Lawrence grade; FTA, femorotibial angle; FIM, functional independence measure; 5mMWS, 5-m maximum walking speed; B: partial regression coefficient, β: standardized partial regression coefficient.

Without confounding factors input: R = 0.568, R2 = 0.323, analysis of variance: p < 0.001.

With confounding factors: R = 0.711, R2 = 0.505, analysis of variance: p < 0.001.

## Discussion

Munin et al. [17] compared groups that began rehabilitation intervention on postoperative days 3 and 7, and they reported that early intervention was effective for reducing the duration of hospitalization. Furthermore, Labraca et al. [18] compared groups that started rehabilitation interventions at 24 hours and 48–72 hours postoperatively, and they found that early intervention reduced the duration of hospitalization. Thus, preliminary research indicates that the early initiation of postoperative rehabilitation can reduce the duration of hospitalization, and it is currently common to begin rehabilitation on postoperative day 1. Nevertheless, it is also important to determine the effect of rehabilitation interventions by clarifying factors that affect the duration of hospitalization for institutions that start rehabilitation on postoperative day 1. Results of the present study indicated that the number of days to independent walking and the magnitude of change in the 5mMWS can be used to assess the effectiveness of early rehabilitation among patients who had undergone MI-TKA. In the current study, the number of days to independent walking was defined using the 3 aforementioned criteria. Of interest, the TUG result can be used as an index for evaluating functional mobility, which includes dynamic balance ability, by evaluating an individual’s standing, sitting, walking, and changes in direction [19], and the optimal TUG cut-off value for predicting falls has been previously reported [14]. However, the definition of independent walking is currently based on the physical therapist’s empirical opinion. Thus, it would be useful to have an objective clinical index, in addition to the physical therapist’s experience and the patient’s self-assessment, to evaluate independent walking. Results of the present study showed that the recovery of walking speed and the number of days to independent walking were important factors that affected the duration of hospitalization. Therefore, evaluation of independent walking, used in this study, may be a useful clinical index for evaluating patients who have undergone MI-TKA and assessing the effectiveness of early rehabilitation.

Remarkably, Kennedy et al. [20] reported that the preoperative TUG result and the 6-minute walking test result were predictors of walking ability at postoperative month 4 in their study of 152 patients who had undergone TKA and total hip arthroplasty. However, their findings were limited because the change in walking ability could not be determined as a predictor of various outcomes (e.g., the duration of hospitalization), and they were not able to evaluate their effects independently from the effect of confounding factors, such as demographic and clinical variables. In contrast, the present study indicated that the recovery of walking speed (as measured using the 5mMWS) and the acquisition of independent walking can be used to assess the effectiveness of early rehabilitation. Therefore, rehabilitation interventions should target improvements in these parameters to reduce the duration of hospitalization.

This study has several limitations. First, we did not investigate the period from the onset of knee OA to TKA and the implementation period of conservative treatment. Second, the study was performed at multiple centers that did not have a unified postoperative treatment policy (e.g., the medication status and timing of the evaluation were different) with the exception of the rehabilitation program. For this reason, it is unclear whether the absence of a unified treatment policy affected the results. The postoperative treatment is carried out by a medical team. Therefore, future studies should assess the effectiveness of early rehabilitation at hospitals having the same postoperative treatment policies.

## Conclusions

Our results indicated that the acquisition of independent walking and the recovery of walking speed can be used to assess the effectiveness of early rehabilitation among patients who have undergone MI-TKA. Thus, patients who experience minimal improvement in their 5mMWS or delayed achievement of independent walking may experience prolonged hospitalization. Therefore, these measures can be used to evaluate the effectiveness of early postoperative rehabilitation for reducing the duration of hospitalization.
